# Evolution of care during pregnancy and childbirth in the extreme south of Brazil

**DOI:** 10.11606/s1518-8787.2021055003128

**Published:** 2021-08-03

**Authors:** Juraci A. Cesar, Raul A. Mendoza-Sassi, Luana P. Marmitt

**Affiliations:** I Universidade Federal do Rio Grande Faculdade de Medicina Programa de Pós-Graduação em Saúde Pública Rio GrandeRS Brasil Universidade Federal do Rio Grande. Faculdade de Medicina. Programa de Pós-Graduação em Saúde Pública. Rio Grande, RS, Brasil; II Universidade do Oeste de Santa Catarina Programa de Pós-Graduação em Biociências e Saúde Flor da SerraSC Brasil Universidade do Oeste de Santa Catarina. Programa de Pós-Graduação em Biociências e Saúde. Flor da Serra, SC, Brasil

**Keywords:** Prenatal Care, trends, Maternal-Child Health Services, Reproductive Health Services, Birthing Centers, methods, Program Evaluation

## Abstract

**OBJECTIVES:**

To describe the evolution of care during pregnancy and childbirth among postpartum women living in the municipality of Rio Grande, Southern Brazil, using data from surveys carried out every three years between 2007 and 2019.

**METHODS:**

Within 48 hours after delivery, a single, standardized questionnaire was applied to all mothers who had children in local hospitals and met the inclusion criteria. Demographic and reproductive characteristics, lifestyle habits, socioeconomic level of the family, and care received during pregnancy and childbirth were investigated. In the analysis, the chi-square test for linear trend was used to assess the distribution of indicators per survey.

**RESULTS:**

A total of 12,645 parturients were interviewed (98% of the women eligible to participate in the surveys). In the period evaluated, the proportion of births fell 35% among adolescents and increased 25% among women aged 35 years and over. Mothers gained, on average, two years of schooling, and their families experienced an important economic improvement, followed by loss of income in the last survey. Maternal smoking, before and during pregnancy, fell by half. The rate of mothers who started prenatal care in the first trimester and the number of consultations and laboratory tests increased. Almost 60% of prenatal consultations and 80% of births took place in the Brazilian Unified Health System. In 2019, vaginal delivery was once again the most common. The rates of low birth weight (9%) and prematurity (17%) virtually remained unchanged.

**CONCLUSIONS:**

We found an important change in the reproductive profile and increased coverage of various prenatal care and delivery services. Children continue to be born well, but low birth weight and prematurity remain endemic.

## INTRODUCTION

Prenatal care and childbirth are one of the main components of maternal and child health^1^ . The care offered on these occasions educates, prevents, cures, and promotes health and well-being in a moment when with increased risk of developing a disease or death^2^ .

In Brazil, the latest national surveys show that between 2011 and 2015 access to prenatal care and hospital birth was nearly universal^3^ . The proportion of mothers who completed seven or more consultations increased from 49% in 1995 to 67% in 2015^3^ . The *Nascer no Brasil* (Birth in Brazil) study (2011–2012) identified that 53% and 69% of Brazilian pregnant women started prenatal care in the first trimester of pregnancy and had at least six prenatal consultations^4^ . The research *Pesquisa Nacional de Saúde de 2013* (PNS - National Health Survey) showed that 71% of pregnant women received adequate prenatal care, starting in the first trimester of pregnancy, attended six or more medical appointments, and underwent at least one blood, urine, and pelvic ultrasound examination^5^ .

Studies carried out in Pelotas (RS) showed that the proportion of mothers who started prenatal care had six or more medical appointments and completed prenatal care adequately increased from 95%, 67%, and 41% in 1982 to 98%, 84%, and 63% in 2015, respectively^6^ . In São Luís (MA), these prevalences increased from 56.5%, 34.8% and 47.3% in 1997/1998 to 67.5%, 60.5%, and 58% in 2010, respectively^7 , 8^ . The data show a substantial improvement in the provision of these services. However, no other time series study evaluating these same indicators has been carried out in Brazil since then.

Between 2007 and 2019, every three years, perinatal surveys were carried out in Rio Grande (RS) to assess care received during pregnancy and childbirth. This series of studies with primary data, capable of providing information not recorded by systems such as the *Sistema de Informações sobre Nascidos Vivos* (Sinasc - Live Birth Information System), had regular periodicity and a unified methodology, including all births that occurred during in Rio Grande, a medium-sized municipality, for a whole year. This type of research, unique in Brazil, allows the assessment of trends in various indicators, from the beginning of pregnancy to the immediate postpartum period. In addition, the period covered included two changes in the command of the Federal Government, which impacted the socioeconomic conditions of the population and the offer and access to prenatal care and childbirth services^9^ .

This article describes the methodology used in the five surveys carried out between 2007 and 2019 and shows how the indicators of assistance during pregnancy and childbirth in the municipality of Rio Grande (RS) evolved during this period.

## METHODS

The perinatal surveys in Rio Grande started in 2007, and since then they have been carried out rigorously every three years: 2007, 2010, 2013, 2016, and 2019. The surveys aim to assess care during pregnancy and childbirth in this municipality, whose population increased from 195,000 to 211,000 inhabitants between 2017 and 2019. The municipality’s public health system, however, remained unchanged, with two hospitals (only one is a fully public hospital), four specialty clinics, and 36 basic health units. The municiaplity had only one maternal death in 2017 and two in 2019. The infant mortality rate increased from 9.3 to 11.6 per 1,000 live births in the same period^10^ .

We included in the study mothers residing in the municipality of Rio Grande who gave birth in the only two local hospitals between January 1st and December 31st of the years in which the survey was carried out. Furthermore, we considered only newborns who had at least 500 grams or 20 weeks of gestational age.

Mothers were approached only once, within 48 hours after delivery, while still in the hospital, which characterizes this design as a cross-sectional study. Trained interviewers applied a single questionnaire.

The questionnaire was divided into blocks from A to I, and aimed to investigate from pregnancy planning to the immediate postpartum period. Block A identified the hospital, the mother, and the newborn. Block B included labor signs and symptoms that took the mother to the hospital, procedures, and guidelines received since hospitalization, opinion on the approach of health professionals, and presence of a companion. Block C evaluated clinical and laboratory exams, the month when prenatal care began, the number of consultations, medication consumption, and morbidity in the current pregnancy. Block D investigated reproductive health, including the number of pregnancies, abortions, offspring, age at first pregnancy and childbirth, and contraceptive methods used. Block E addressed lifestyle and behavior habits, including sociodemographic characteristics of the mother, smoking, physical exercise, depression, and alcohol, coffee, and mate consumption. Block F investigated the socioeconomic characteristics and occupation of family members, asking about the household’s income in the month prior to the interview. In Block G, the information contained in the Pregnant Woman’s Card was reproduced, and in Block H, the measurements of the newborn’s physical examination. Finally, in Block I, data were collected for further contact and, if necessary, visit the mother and newborn.

“Family income” refers to the amount received, from any source, in the month prior to the interview by the household members. Birthweight when less than 2,500 grams was defined as low. We classified birth with a gestational age assessed by ultrasound or with a date of last menstruation less than 37 weeks as premature. Mothers who smoked at least one cigarette a day in the last 30 days were classified as smokers. “Hospitalization” refers to a hospital stay of at least 24 hours.

Each survey had four interviewers who were trained and participated in a pilot study. All interviewers were graduates of humanities or biological sciences. Two of the interviewers visited the maternity hospitals daily throughout the week and applied the questionnaire, while on weekends this task was performed by a third interviewer. The fourth interviewer assisted in the interviews and made home visits when mothers left the hospital before the mandatory 48 hours.

Every day, the interviewers checked the births that took place the day before in each maternity hospital, visited the wards, and listed the births that took place. The purpose of the study was explained to the mothers. Next, if they agreed to participate, signed two copies of the Informed Consent Form, and maintained one in their possession.

In 2007, 2010, and 2013 surveys, a physical questionnaire was used. The open questions were coded by the interviewers and the questionnaires were revised. If a difference was found, the mothers were contacted again by phone or visited at their homes. After this check, the questionnaires were typed twice, by different professionals, in reverse order^11^ . For each block of 100 questionnaires, these entries were compared and any differences corrected^12^ . In the 2016 and 2019 surveys, data entry was performed simultaneously with the interview, with tablets, and the Research Electronic Data Capture (REDCap)^13^ app. At the end of each day, the questionnaires were downloaded from the Federal University of Rio Grande (FURG) server and revised. At the end of the process, all variables and their categories were labeled.

Preliminary analysis checked for outliers, categorized, and created derived variables. Next, the distribution of the variables of interest was verified according to the year of the perinatal survey, using the chi-square test for linear trends. We also estimated the central tendency and dispersion measures. We performed all analyzes in the Stata 11.0 statistical package^14^ .

For quality control, around 10% of the interviews were partly redone by telephone. For these cases, a set of questions equal to those asked at the hospital was reapplied within 15 days after hospital discharge. To assess the agreement in the answers provided by the mothers, we used the Kappa index, which ranged from 0.60 (pregnancy planning) to 0.99 (type of delivery). Most responses ranged from 0.72 to 0.91, indicating a good level of agreement.

All research protocols were approved by the FURG Health Research Ethics Committee under the following numbers: 2007 survey (05369/2006), 2010 survey (06258/2009), 2013 survey (02623/2012), 2016 survey (0030-2015), and 2019 survey (278/2018).

## RESULTS

12,946 births met the research inclusion criteria in the years the survey was carried out. In total, we were able to collect information from 12,645 births, representing 98% of the total.

Table 1 shows that over 12 years, the occurrence of childbirths among adolescents reduced by 35%, while it increased 25% among women aged 35 years and over. Mothers presented a gain of two years of schooling in the same period. The participation of mothers in the labor market increased by 14%. Family income also increased, and the unemployment rate fell until 2013. The year 2016 indicates an opposite trend, but among the poorest (< 1SM), part of the improvement in income persisted in 2019. Throughout the period, 10% of mothers remained as the person with the highest income in the household.

**Table 1:** Main characteristics of mothers and families included in perinatal studies in Rio Grande (RS), Brazil, 2007–2019.

Characteristics	2007	2010	2013	2016	2019	2007–2019	p trend
Mother's age (years)							
> 20	20.2%	18.6%	17.3%	16.9%	13.1%	-35.1%	< 0.001
20 to 24	28.1%	26.8%	26.3%	26.1%	27.2%	-3.2%	0.353
25 to 29	24.6%	25.9%	24.1%	23.6%	23.4%	-4.9%	0.100
30 to 34	15.4%	18.6%	19.8%	19.9%	20.6%	+25.2%	< 0.001
35+	11.8%	10.1%	12.4%	13.4%	15.7%	+24.8%	< 0.001
Mean (standard deviation)	25.6 (6.6)	25.9 (6.4)	26.3 (6.5)	26.5 (6.6)	27.1 (6.6)	+5.9%	-
Median	25	25	26	26	26	+4.0%	-
skin color							
White	69.5%	69.4%	66.1%	67.0%	76.5%	+10.1%	< 0.001
Brown	18.3%	20.6%	22.3%	22.6%	15.0%	-18.0%	0.156
Black	12.2%	9.9%	11.7%	10.3%	8.5%	-30.3%	< 0.001
The mother lived with her husband/partner	82.8%	83.2%	85.8%	83.6%	85.1%	+1.6%	0.034
Mother's education (years)							
0	0.9%	0.4%	0.0%	0.1%	0.1%	-88.9%	< 0.001
1 to 4	11.7%	7.6%	6.0%	3.3%	4.1%	-65.0%	< 0.001
5 to 8	36.1%	37.2%	33.6%	33.4%	27.3%	-24.4%	< 0.001
9 to 11	41.9%	44.5%	44.7%	39.8%	46.9%	+11.9%	0.139
12+	9.4%	10.3%	15.6%	23.5%	21.6%	+129.8%	< 0.001
Mean (standard deviation)	8.6 (3.5)	9.0 (3.2)	9.5 (3.3)	10.1 (3.6)	10.5 (3.9)	+22.1%	-
Median	9	10	10	11	11	+22.2%	-
Had paid work during pregnancy (n = 12,136)	37.4%	42.8%	43.6%	45.9%	42.6%	+13.6%	< 0.001
Monthly family income in minimum wages (MW) (n = 12,184)							
< 1	12.4%	9.6%	3.4%	5.9%	9.9%	-20.2%	< 0.001
1.0 to 1.9	33.4%	37.1%	29.1%	31.4%	34.6%	+3.6%	0.295
2.0 to 3.9	34.7%	34.2%	40.2%	40.6%	40.1%	+15.6%	0.077
≥ 4.0	19.6%	19.1%	27.3%	22.1%	15.4%	-21.4%	< 0.001
Mean (standard deviation) in MW	2.9 (3.2)	3.2 (3.9)	3.4 (2.9)	3.2 (3.1)	2.6 (2.3)	-10.3%	-
Median in MW	2.1	2.2	2.6	2.3	2.0	-4.8%	-
Number of residents in the household							
Mean (standard deviation)	3.7 (1.8)	3.5 (1.8)	3.4 (1.7)	3.3 (1.6)	3.5 (1.5)	-5.4%	-
Median	3	3	3	3	3	0.0%	-
The person with higher income was unemployed (n = 12,136)	15.2%	10.5%	7.3%	16.8%	15.1%	-0.7%	0.008
The woman was the person with a higher income	8.8%	11.1%	9.9%	11.5%	9.0%	+2.3%	0.169

Total	n	2,557	2,395	2,687	2,694	2314	
		20.2	18.9	21.2	21.3	18.3	

Table 2 shows a drop of approximately 50% in the prevalence of smoking during pregnancy and in the preceding six months between 2007 and 2019.

**Table 2:** Reproductive characteristics, lifestyle, and morbidity during the gestational period among parturients included in perinatal studies in Rio Grande (RS), Brazil, 2007–2019.

Characteristics		2007	2010	2013	2016	2019	2007–2019	p trend
Primiparous		39.5%	43.4%	47.3%	43.1%	38.1%	-3.5%	0.457
Parity								
Mean (standard deviation)		2.1 (1.4)	2.0 (1.3)	1.8 (1.0)	2.2 (1.3)	2.1 (1.3)	0.0%	-
Median		2	2	2	2	2	0.0%	-
The mother has already had a stillborn child.		3.5%	3.5%	1.7%	2.8%	2.2%	-37.1%	0.003
The mother reported the occurrence of spontaneous or induced abortion in the past		19.2%	13.8%	15.3%	15.3%	15.2%	-20.8%	0.018
The mother smoked at least one cigarette a day in the six months prior to pregnancy		27.8%	26.3%	22.5%	15.1%	14.1%	-49.3%	< 0.001
The mother smoked at least one cigarette a day during at least one of the trimesters of pregnancy		23.1%	20.8%	18.7%	12.7%	12.5%	-45.9%	< 0.001
Morbidity in the gestational period								
Systemic arterial hypertension		18.0%	19.6%	19.6%	18.2%	12.5%	-30.6%	< 0.001
Diabetes mellitus		2.9%	3.1%	5.1%	4.5%	9.3%	+220.7%	< 0.001
Anemia		51.7%	41.5%	36.6%	31.2%	37.8%	-26.9%	< 0.001
Depression		16.9%	10.2%	9.9%	3.6%	2.2%	-87.0%	< 0.001
Pathological vaginal discharge		52.0%	42.9%	43.4%	32.5%	21.7%	-37.5%	< 0.001
Maternal hospitalizations		13.4%	10.7%	8.9%	8.9%	5.3	-60.4%	< 0.001

Total	n	2.557	2.395	2.687	2.694	2314		
	%	20.2	18.9	21.2	21.3	18.3		

Table 3 shows that prenatal care improved substantially over the period. More women started prenatal care in the first trimester of pregnancy, and the number of medical appointments also increased. The vast majority of mothers attended at least six medical appointments and underwent two serological tests for HIV, syphilis, and urine. However, the rate of cytopathological examination of the uterine cervix and clinical examination of the breasts is still low. In 2019, vaginal delivery was once again the most common. The occurrence of episiotomy dropped from 71% to 19%, and delivery care by a physician was nearly universal. Finally, 58% of the parturients attended all the prenatal medical consultations, and 76% delivered in the Brazilian Unified Health System (SUS).

**Table 3:** Prenatal care and childbirth among postpartum women included in perinatal studies in Rio Grande (RS), Brazil, 2007–2019.

Characteristics		2007	2010	2013	2016	2019	2007–2019	p trend
Planned pregnancy		37.0%	36.0%	37.5%	39.4%	32.8%	-11.4%	< 0.001
Held prenatal		95.8%	95.5%	97.4%	98.5%	96.7%	+3.4%	< 0.001
The mother attended six or more medical appointments		72.5%	76.7%	83.5%	84.3%	85.7%	+18.2%	< 0.001
Number of prenatal consultations performed								
Mean (standard deviation)		7.4 (3.7)	7.7 (3.6)	8.3 (3.3)	8.2 (3.1)	8.7 (3.4)	10.8%	
Median		7	8	8	8	9	14.3%	-
Consultations started in the 1st trimester of pregnancy		73.6%	78.3%	78.6%	79.4%	81.5%	+10.7%	< 0.001
Place of prenatal care								
Basic Health Unit		41.0%	33.5%	29.7%	36.4%	49.2%	+20.0%	< 0.001
Outpatient		20.0%	24.3%	22.0%	20.7%	14.6%	-27.0%	< 0.001
Private/Medical insurance		39.0%	42.1%	48.3%	42.9%	36.2%	-7.2%	0.225
During prenatal consultations, the mother performed:								
2+ HIV serological tests		67.7%	61.4%	75.8%	84.4%	93.6%	+38.3%	< 0.001
2+ serological tests for syphilis		27.0%	56.2%	73.5%	78.4%	87.4%	+223.7%	< 0.001
2+ qualitative urine tests		70.8%	72.1%	76.8%	75.9%	85.0%	+117.9%	< 0.001
1+ abdominal ultrasound		92.3%	92.8%	94.9%	98.4%	93.3%	+1.1%	< 0.001
The mother underwent cytological examination of the cervix		37.5%	37.8%	45.7%	30.9%	57.6%	+53.6%	< 0.001
The mother had the breast examined		47.1%	56.2%	49.2%	39.9%	65.3%	+38.6%	< 0.001
The mother were immunized against tetanus		78.5%	79.0%	82.5%	78.1%	85.4%	+8.8%	< 0.001
The mother received ferrous sulfate		59.0%	75.6%	78.8%	80.1%	81.1%	+37.5%	< 0.001
The mother started prenatal visits in the first trimester, had six or more visits and at least two HIV, syphilis, and qualitative urine tests (n = 8,828)		18.1%	40.2%	51.6%	37.4%	64.0%	+253.6%	< 0.001
Delivery type								
Vaginal delivery		48.4%	43.4%	38.5%	45.8%	50.5%	+4.5%	0.069
Caesarean		51.6%	56.6%	61.5%	54.2%	49.5%	-4.1%	
Use of forceps		7.3%	9.2%	7.7%	4.7%	2.0%	-72.6%	< 0.001
Episiotomy (n = 5,715)		70.9%	68.0%	60.2%	40.1%	19.4%	-70.9%	< 0.001
Delivery attended by a physician		85.3%	92.4%	95.3%	97.1%	98.8%	+15.8%	< 0.001
The birth took place in SUS		79.1%	75.9%	65.5%	76.2%	83.9%	+6.1%	0.001

Total	n	2,557	2,395	2,687	2,694	2314		
	%	20.2	18.9	21.2	21.3	18.3		

Table 4 shows that the vast majority of children are delivered with adequate weight (91%) and at term (83%). Few newborns require hospitalization (6%), and less than a quarter have an important risk factor (22%). Low birth weight (< 2,500 g) and prematurity (< 37 weeks of gestation) affected 9% and 17% of newborns, respectively. Just over half (56%) of the mothers were admitted to the hospital carrying the Pregnant Woman’s Card.

**Table 4:** Main characteristics of newborns included in perinatal studies in Rio Grande (RS), Brazil, 2007–2019.

Characteristics		2007	2010	2013	2016	2019	2007–2019	p trend
Sex								
Male		51.2%	51.0%	52.4%	51.0%	51.0%	+0.4%	0.907
Feminine		48.8%	49.9%	47.6%	49.0%	49.9%		
Single birth newborns		98.7%	98.3%	97.5%	98.3%	98.1%	-0.6%	0.166
Live birth		98.5%	99.1%	98.8%	99.1%	99.4%	+0.9%	0.005
Birth weight (grams)								
≤ 2,499		9.4%	8.9%	9.7%	8.8%	10.1%	+7.4%	0.533
2,500–2,999		22.8%	24.3%	23.0%	21.6%	20.8%	-8.8%	0.015
3,000–3,499		40.6%	39.2%	40.0%	40.7%	40.1%	-1.2%	0.866
3,500–4,999		21.5%	21.8%	21.9%	22.5%	23.1%	+7.4%	0.146
≥ 5,000		5.7%	5.8%	5.4%	6.4%	5.8%	+1.8%	0.510
Mean (standard deviation), grams		3,172 (594)	3,184 (577)	3,171 (588)	3,203 (569)	3,190 (590)	+5.7%	-
Median		3.205	3.220	3.205	3.275	3.230	+7.8%	-
Gestational age (weeks) (n = 10,825)								
20–33.9		6.0%	5.2%	7.2%	5.4%	7.1%	+18.3%	0.210
34–36.9		12.2%	10.5%	11.4%	10.1%	10.0%	-18.0%	0.023
37–39.9		48.0%	49.9%	50.1%	48.3%	48.8%	+1.7%	0.917
40+		33.8%	34.3%	31.2%	36.1%	34.1%	+0.9%	0.474
Mean (standard deviation), grams		38.7 (3.1)	38.8 (3.0)	38.5 (3.1)	38.9 (3.0)	38.6 (3.2)	+0.1%	-
Median (grams)		39.1	39.1	40.3	39.3	39.3	-0.2%	-
Required hospitalization		6.4%	5.5%	7.0%	6.1%	6.1%	-4.7%	0.792
Newborns with low birth weight, premature, or required hospitalization (n = 10,825)		23.6%	21.4%	24.7%	20.6%	21.5%	-8.9%	0.075
Mothers who had a Pregnant Card when they were hospitalized for childbirth		54.7%	51.3%	59.1%	53.5%	60.8%	+11.2%	< 0.001

Total	n	2,557	2,395	2,687	2,694	2314		
	%	20.2	18.9	21.2	21.3	18.3		

## DISCUSSION

The data show an improvement in the socioeconomic condition of the families and, mainly, in the care during pregnancy and childbirth in the municipality of Rio Grande between 2007 and 2019. During this period, mothers gained two years of schooling, increased, and maintained participation in the labor market, delayed the pregnancy age, and significantly quit smoking. The children were born in good health conditions but continue to live with high rates of low birth weight and, above all, prematurity. Although in 2016 some indicators stopped improving or even got worse, pregnancy and childbirth care in 2019 are much higher than in 2007.

Adolescent births in Rio Grande fell 35% in the period, while they increased 25% among women aged 35 and over. In Brazil, the adolescent birth rate fell from 36% in 2000 to 24% in 2017^15^ . In Ribeirão Preto (SP), from 29.3% in 1997/1998 to 18.5% in 2010^7^ , and in Pelotas from 15.4% in 1982 to 14.6% in 2015^6^ . Regarding the proportion of births among women aged 35 or over, the increase observed in Rio Grande is similar to the increase in Pelotas, from 9.9% in 1982 to 14.8% in 2015^6^ . In the country as a whole, this data has changed little. In 1996, 9.9% of the parturients were aged 35 or over^16^ , reaching 10.5% in 2011/2012^5^ and 11.8% in 2012/2013^17^ .

The decrease in the occurrence of pregnancy among teenagers, as well as the increase among older women, can be mainly attributed to greater access to health services^17^ , the increase in the level of education^18^ , the drop in the fertility rate^3 ,^ and the greater insertion of women in the labor market, especially in Rio Grande, as a result of the naval industry^19^ .

Several studies show a substantial improvement in pregnancy and childbirth care in Brazil in the last three or four decades^3 , 6 , 7 , 9^ . The turning point for this change was the implementation of SUS in 1989^20^ . Since then, several programs have been implemented, with emphasis on the current *Estratégia Saúde da Família* (ESF - Family Health Strategy)^21^ .

In Rio Grande, access to prenatal care changed little in the period, remaining at 97%, but the provision of prenatal care considered to be minimally adequate increased from 18.1% in 2007 to 63.2% in 2019. In Pelotas, this indicator went from 41% in 1982 to 63% in 2015^6^ , and in São Luís, from 47.3% in 1997/1998 to 58.2% in 20108. In Brazil as a whole, it increased from 15%^17^ to 71.4%^5^ . However, it should be noted that the comparison of this specific indicator with other studies is hampered due to the use of different criteria.

The improvement observed in Rio Grande is due to the expansion of primary care coverage, which went from 55.3% in 2007 to 87.5% in 2019. This growth was due to the increase in the number of ESF teams and the greater offer of services in the city’s ghettos and rural areas. However, increasing the proportion of mothers with adequate prenatal care is still a major challenge in the municipality. A continuing need to increase the offer of clinical breast examination, cervical cytopathological examination, immunization against neonatal tetanus, and supplementation with ferrous sulfate are also necessary. Although 80% of all pregnant women started prenatal care in the first trimester and had six or more medical appointments, the coverage for the procedures mentioned above was around 60%, which denotes a loss of opportunity for intervention.

The occurrence of cesarean sections increased from 51.6% in 2007 to 61.5% in 2013 but dropped to 49.5% in 2019. Although the drop in the entire period was only 4%, between 2016 and 2019 this reduction was 20%, which shows a clear trend towards a reduction in the use of the procedure in the municipality. In Brazil, this occurrence increased from 40.2% in 1995 to 55.5% in 2015^3^ . In Pelotas, it increased from 27.6% in 1982 to 64.9% in 2015^6^ , while in São Luís it increased from 34.1% in 1997/1998 to 47.5% in 2010^7^ .

The reduction in cesarean sections in Rio Grande, despite the absence of interventions discouraging the procedure, is due to the partial and temporary closure of one of the local hospitals, which met all the demands of the private sector. The hospital had a frequency of cesarean sections that were 50% higher compared to the hospital dedicated exclusively to SUS patients. It is noteworthy, however, that the proportion of cesarean sections in the municipality is still three times higher than that recommended as reasonable by the World Health Organization (WHO), including high-risk pregnancies^22^ .

The occurrence of episiotomy in Rio Grande fell from 70.9% in 2007 to 19.4% in 2019. Brazil lacks population-based studies evaluating trends on the use of episiotomy, hindering comparisons. The Birth in Brazil study found an index of 56.1% for the country as a whole, ranging from 48.6% in the North region to 69.2% in the Midwest, and from 55.5% in the public sector to 67.1% in the private sector^23^ .

In addition to the lack of clinical evidence of the need for episiotomy, its occurrence leads to bleeding, lesions in the perineal region, sphincter trauma, fecal incontinence, and prolonged postpartum pain, among other complications^24^ . Despite this, the WHO recommends that an acceptable episiotomy rate should be around 10%^22^ . The high rates observed in Brazil result from the fact that births are performed mainly by physicians, whose practice is characterized by an excess of obstetric interventions^25 , 26^ .

Similar to policies on cesarean sections, Rio Grande also lacked specific interventions aimed at reducing episiotomies. The changes that occurred during the period may be related to the obligation, established by the Ministry of Health, that this procedure is performed with the woman’s authorization, the presence of a companion in the pre-delivery period, and the greater participation of the nursing area. The partial closure of the other hospital, where the frequency of episiotomy was higher, must also have contributed to this very sharp decrease.

The proportions of low birth weight and prematurity in Rio Grande rose from 9% and 18% in 2007 to 10% and 17% in 2019. In Pelotas, the prevalence of low birth weight increased from 9% in 1982 to 10% in 2015, while prematurity increased from 6% to 14%^26^ . In São Luís, the prevalence of low birth weight and prematurity remained virtually stable between 1997/1998 and 2010, at around 8% and 13%, respectively^7^ . In Brazil as a whole, the occurrence of low birth weight and prematurity remained between 1995 and 2015 at 8% and 11%, respectively^3^ .

Considering the improvements that have taken place since the implementation of the SUS in 1989 – expansion of the basic health network, the programs *Estratégia Saúde da Família, Rede Cegonha* , and *Mais Médicos* , among other initiatives –, the only possible explanation for the absence of a decrease (or even an increase in some locations) is the excess of cesarean sections^26^ . In Rio Grande, the proportion of C-sections among mothers in the highest income quintile is 74.4% against 41.1% in the worst quartile.

The prevalence of smoking before and during pregnancy dropped by about 50% in the period. A similar trend was observed in Pelotas (from 35.7% in 1982 to 16.5% in 2015) and Ribeirão Preto (from 28.8% to 11.8%)^27^ . In Pelotas, this decrease occurred mainly among white and high-income women^28^ In the country as a whole, it decreased from 15.6% in 2006 to 10.8% in 2014^29^ .

Smoking is harmful to the health of the fetus because it restricts intrauterine growth and increases the chances of prematurity and low birth weight^30^ . Public anti-smoking policies should prioritize mothers with greater social vulnerability. If measures are not adopted, smoking will continue to damage maternal and child health, with sequelae in adulthood^28^ .

The use of the pregnant woman’s card at the time of delivery increased from 54.7% to 60.8% in Rio Grande. None of the studies mentioned above published data on this indicator. The *Birth in Brazil* study found a prevalence of ownership at hospital admission of 74.6% for Brazil as a whole, ranging from 46% in the Midwest region to 83.6% in the South region^4^ .

The use of the Pregnant Woman’s Card is usually low because it is an undervalued document. Efforts are required to make mothers aware of the need to take this card to all appointments, and professionals must fill out the document adequately and completely, unlike what has been done^31^ . The correct use of the Pregnant Woman’s Card would optimize the provision of care to pregnant women and newborns.

The worsening of family income observed since 2016 is basically due to the reduction in the supply of jobs in the naval sector in Rio Grande. The assembly of oil platforms generated, between 2007 and 2008 and 2015 and 2016, around 30 thousand direct and indirect jobs in the region^19^ , occupying all the surplus labor in Rio Grande and neighboring municipalities. In 2019, however, the same sector generated just over a dozen jobs.

The perinatal studies in Rio Grande are perhaps the only source of primary data collected regularly, at short intervals, and for a relatively long period, in a medium-sized Brazilian municipality. The same methodology was used in the five years of investigation, and the rate of respondents was high, including almost all births in both the urban and rural areas of the municipality. The only limitation of the study is the fact that it is based almost exclusively on information provided by the mother, with the possibility of recall bias. This, however, does not make the results unfeasible, as the studies used here for comparison have the same issue.

The series of surveys showed that practically all indicators of assistance during pregnancy and childbirth have been improving in the municipality of Rio Grande. In quantitative terms, the care received in 2019 is undeniably higher than in 2007. However, it should be noted that the universalization of the provision of adequate care, both during prenatal care and at the time of delivery, is still far short of what is desired. Evidence still points to unnecessary interventions and lost opportunities. The local health team needs to make efforts to ensure the pregnant woman all the necessary care. Optimizing the provision of this care is essential to reduce maternal and child morbidity and mortality in the municipality. Finally, the importance of the SUS stands out, as the place where most prenatal consultations and births take place. Strengthening it is a matter of social justice in times as dark and uncertain as those Brazil has been going through.
