# Supplementary material for: Evolution of care during pregnancy and childbirth in the extreme south of Brazil
Source: Rev Saude Publica. 2021 Aug 3;55:50. doi: 10.11606/s1518-8787.2021055003128 (PMC8328512; doi:10.11606/s1518-8787.2021055003128)
Supplement: Translation

# Translation: Evolução da assistência à gestação e ao parto no extremo sul do Brasil

**Authors:** Juraci A. Cesar, Raul A. Mendoza-Sassi, Luana P. Marmitt

**Affiliations:** I Brasil Universidade Federal do Rio Grande. Faculdade de Medicina. Programa de Pós-Graduação em Saúde Pública. Rio Grande, RS, Brasil; II Brasil Universidade do Oeste de Santa Catarina. Programa de Pós-Graduação em Biociências e Saúde. Flor da Serra, SC, Brasil

**Keywords:** Cuidado Pré-Natal, tendências, Serviços de Saúde Materno-Infantil, Serviços de Saúde Reprodutiva, Centros de Assistência à Gravidez e ao Parto, métodos, Avaliação de Programas e Projetos de Saúde

## Abstract

**OBJETIVO:**

Descrever a evolução da assistência à gestação e ao parto entre puérperas residentes no município de Rio Grande (RS) utilizando dados de inquéritos realizados a cada três anos, entre 2007 e 2019.

**MÉTODOS:**

Em até 48 horas após o parto foi aplicado questionário único, padronizado, a todas as mães que tiveram filhos nos hospitais locais e cumpriram os critérios de inclusão. Foram investigadas características demográficas e reprodutivas, hábitos de vida, nível socioeconômico da família e cuidados recebidos durante a gestação e o parto. Na análise, utilizou-se o teste qui-quadrado de tendência linear para avaliar a distribuição dos indicadores por inquérito.

**RESULTADOS:**

Ao todo, 12.645 parturientes foram entrevistadas (98% do total de mulheres aptas a participar da pesquisa). No período avaliado, a proporção de partos caiu 35% entre adolescentes e aumentou 25% entre mulheres com 35 anos ou mais. As mães ganharam, em média, dois anos de escolaridade, e suas famílias tiveram importante melhora econômica, seguida, porém, de perda de renda no último inquérito. O tabagismo materno, antes e durante a gravidez, caiu à metade. Houve aumento na taxa de mães que iniciaram o pré-natal no primeiro trimestre, e aumentou também o número de consultas e de testes laboratoriais. Quase 60% das consultas de pré-natal e 80% dos partos ocorreram no Sistema Único de Saúde. Em 2019, o parto vaginal voltou a ser o mais comum. As taxas de baixo peso ao nascer (9%) e prematuridade (17%) praticamente não se modificaram.

**CONCLUSÕES:**

Houve mudança importante no perfil reprodutivo e aumento da cobertura de diversos serviços de assistência pré-natal e parto. As crianças seguem nascendo bem, mas o baixo peso ao nascer e a prematuridade continuam endêmicos.

## INTRODUÇÃO

A assistência pré-natal e ao parto é um dos principais componentes da saúde materno-infantil^1^ . Os cuidados oferecidos nessas ocasiões educam, previnem, curam e promovem saúde e bem-estar em um período em que há risco elevado de adoecer e morrer^2^ .

No Brasil, os últimos inquéritos nacionais apontam que, entre 2011 e 2015, o acesso ao pré-natal e ao parto hospitalar era praticamente universal^3^ . A proporção de mães que completaram sete ou mais consultas foi de 49% em 1995 para 67% em 2015^3^ . O estudo Nascer no Brasil (2011–2012) identificou que 53% e 69% das gestantes brasileiras iniciavam o pré-natal no primeiro trimestre da gravidez e passavam por pelo menos seis consultas de pré-natal^4^ . A Pesquisa Nacional de Saúde (PNS) de 2013 mostrou que 71% das gestantes receberam pré-natal adequado, com início no primeiro trimestre da gestação, seis ou mais consultas e ao menos um exame de sangue, urina e ultrassom pélvico^5^ .

Estudos conduzidos em Pelotas (RS) mostraram que a proporção de mães que iniciaram o pré-natal, passaram por seis ou mais consultas e completaram o pré-natal adequadamente aumentou de 95%, 67% e 41% em 1982 para 98%, 84% e 63% em 2015, respectivamente^6^ . Em São Luís (MA), essas prevalências subiram de 56,5%, 34,8% e 47,3% em 1997/1998 para 67,5%, 60,5% e 58% em 2010, respectivamente^7 , 8^ . Os dados mostram substancial melhora na oferta desses serviços. No entanto, desde então, nenhum outro estudo de série temporal foi conduzido no Brasil para avaliar esses mesmos indicadores.

Entre 2007 e 2019, a cada três anos, foram realizados em Rio Grande (RS) inquéritos perinatais que avaliam a assistência à gestação e ao parto. Essa série de estudos com dados primários, capaz de fornecer informações não coletadas por sistemas como o Sistema de Informações sobre Nascidos Vivos (Sinasc), teve periodicidade regular e metodologia unificada, incluindo todos os nascimentos ocorridos em Rio Grande, município de porte médio, durante um ano inteiro. Esse tipo de pesquisa, única no Brasil, consegue avaliar tendências de vários indicadores, do início da gestação ao pós-parto imediato. Além disso, o período coberto incluiu duas trocas no comando do Governo Federal, as quais impactaram as condições socioeconômicas da população e a oferta e acesso a serviços de assistência pré-natal e parto^9^ .

O presente artigo descreve a metodologia utilizada nos cinco inquéritos realizados entre 2007 e 2019 e mostra como evoluíram os indicadores de assistência à gestação e ao parto no município de Rio Grande (RS) nesse período.

## MÉTODOS

Os inquéritos perinatais de Rio Grande foram iniciados em 2007 e desde então vêm sendo realizados rigorosamente a cada três anos: 2007, 2010, 2013, 2016 e 2019. O principal objetivo é avaliar a assistência à gestação e ao parto nesse município, cuja população passou de 195 mil para 211 mil habitantes entre 2017 e 2019. A rede pública de saúde do município, no entanto, pouco se modificou, permanecendo com dois hospitais (somente um deles totalmente público), quatro ambulatórios de especialidades e 36 unidades básicas de saúde. Em 2007, houve um óbito materno no município, e em 2019, dois. O coeficiente de mortalidade infantil aumentou de 9,3 para 11,6 por 1.000 nascidos vivos no período^10^ .

Foram incluídas nos estudos mães residentes no município de Rio Grande que deram à luz nos dois únicos hospitais da cidade entre 1º de janeiro e 31 de dezembro dos anos em que o inquérito foi realizado. Além disso, o recém-nascido deveria ter alcançado pelo menos 500 gramas ou 20 semanas de idade gestacional.

As mães foram abordadas uma única vez, em até 48 horas após o parto, ainda no hospital, o que caracteriza este delineamento como do tipo transversal. Nessa ocasião, entrevistadores treinados aplicavam um questionário único.

O questionário, dividido em blocos de A até I, buscava investigar desde o planejamento da gravidez até o pós-parto imediato. O bloco A identificava o hospital, a mãe e o recém-nascido. O bloco B incluía sinais e sintomas que levaram a mãe ao hospital, procedimentos e orientações recebidas desde a hospitalização, opinião sobre a abordagem dos profissionais de saúde e presença de acompanhante. O Bloco C avaliou exames clínicos e laboratoriais, mês de início do pré-natal, número de consultas, consumo de medicamentos e morbidade na gestação atual. O Bloco D investigou saúde reprodutiva, incluindo quantidade de gravidezes, abortos, filhos, idade na primeira gravidez e parto e métodos contraceptivos utilizados. O Bloco E tratou de hábitos de vida e comportamento, incluindo características sociodemográficas da mãe, tabagismo, consumo de álcool, café e chimarrão, exercício físico e depressão. O Bloco F investigava características socioeconômicas e ocupação dos membros da família, perguntando pela renda dos moradores do domicílio no mês anterior à entrevista. No Bloco G foram copiadas informações constantes na Carteira da Gestante, e no Bloco H as medidas do exame físico do recém-nascido. Por fim, no Bloco I, foram coletados dados para posterior contato e, se necessário, visita à mãe e ao recém-nascido.

“Renda familiar” referia-se ao valor recebido, de qualquer fonte, no mês anterior à entrevista pelos moradores do domicílio. O peso de nascimento quando inferior a 2.500 gramas foi definido como baixo. Considerou-se prematuro o nascimento com idade gestacional, avaliada por ultrassonografia ou data da última menstruação, inferior a 37 semanas. Foram classificadas como tabagistas as mães que fumaram pelo menos um cigarro por dia nos últimos 30 dias. E para “hospitalização” considerou-se um período de internação de pelo menos 24 horas.

Cada inquérito contou com quatro entrevistadoras que foram treinadas e participaram de estudo-piloto, todas graduadas em ciências humanas ou biológicas. Duas das entrevistadoras visitavam as maternidades ao longo da semana, diariamente, e aplicavam o questionário, enquanto nos finais de semanas isso era feito por uma terceira entrevistadora. A quarta entrevistadora auxiliava nas entrevistas e fazia visita domicíliar quando as mães deixavam o hospital antes das 48 horas obrigatórias.

Diariamente, as entrevistadoras checavam os nascimentos ocorridos no dia anterior em cada maternidade, visitavam as enfermarias e listavam os partos ocorridos. O objetivo do estudo era explicado às mães, que, caso concordassem em participar, assinavam o termo de consentimento livre e esclarecido em duas vias, ficando uma em sua posse.

Nos inquéritos de 2007, 2010 e 2013, utilizou-se questionário físico. As questões abertas foram codificadas pelas entrevistadoras e os questionários revisados. Havendo diferença, as mães eram contatadas novamente por telefone ou visitadas em seus domicílios. Após essa conferência, os questionários eram digitados duas vezes, por diferentes profissionais, na ordem inversa^11^ . A cada bloco de 100 questionários, essas digitações eram comparadas e eventuais diferenças corrigidas^12^ . Nos inquéritos de 2016 e 2019, a entrada de dados foi feita em simultâneo com a entrevista, com *tablets* e o aplicativo Research Electronic Data Capture (REDCap)^13^ . Ao final de cada dia, os questionários eram descarregados no servidor da Universidade Federal do Rio Grande (FURG) e revisados. No fim do processo, todas as variáveis e suas categorias foram rotuladas.

A análise preliminar conferiu valores muito discrepantes categorizou e criou variáveis derivadas. Em seguida, verificou-se a distribuição das variáveis de interesse conforme o ano do inquérito perinatal, utilizando-se o teste qui-quadrado de tendência linear. Foram calculadas medidas de tendência central e dispersão. Todas as análises foram realizadas por meio do pacote estatístico Stata 11.0^14^ .

Para controle de qualidade, cerca de 10% das entrevistas foram em parte refeitas por telefone. Nesses casos, um conjunto de perguntas iguais àquelas feitas no hospital era reaplicado em até 15 dias após alta hospitalar. O objetivo era avaliar a concordância nas repostas fornecidas pelas mães. Para isso, utilizou-se o índice Kappa, que variou de 0,60 (planejamento da gravidez) a 0,99 (tipo de parto), mas com a maioria das respostas variando de 0,72 a 0,91 – nível de concordância considerado satisfatório.

Todos os protocolos de pesquisa foram aprovados pelo Comitê de Ética em Pesquisa na Área da Saúde da FURG sob os seguintes números: inquérito de 2007 (05369/2006), de 2010 (06258/2009), de 2013 (02623/2012), de 2016 (0030-2015) e de 2019 (278/2018).

## RESULTADOS

Nos anos de realização do inquérito, 12.946 nascimentos cumpriram os critérios de inclusão da pesquisa. Ao todo, foi possível coletar informações de 12.645 nascimentos, 98% do total.

A Tabela 1 mostra que, ao longo de 12 anos, houve redução de 35% na ocorrência de partos entre adolescentes, ao mesmo tempo em que houve aumento de 25% entre mulheres com 35 anos ou mais de idade. Quanto à escolaridade, houve ganho de dois anos no período. A participação das mães no mercado de trabalho aumentou em 14%. A renda familiar também aumentou, e a taxa de desemprego caiu até 2013. Em 2016, observa-se caminho inverso, mas, entre os mais pobres (< 1SM), parte da melhoria na renda ainda persistia em 2019. Em todo o período, 10% das mães se mantiveram como pessoa de maior renda no domicílio.

**Tabela 1:** Principais características das mães e famílias incluídas nos estudos perinatais de Rio Grande (RS), 2007–2019.

Característica	2007	2010	2013	2016	2019	Variação 2007–2019	p-valor tendência
Idade da mãe (anos)							
> 20	20,2%	18,6%	17,3%	16,9%	13,1%	-35,1%	< 0,001
20 a 24	28,1%	26,8%	26,3%	26,1%	27,2%	-3,2%	0,353
25 a 29	24,6%	25,9%	24,1%	23,6%	23,4%	-4,9%	0,100
30 a 34	15,4%	18,6%	19.8%	19,9%	20,6%	+25,2%	< 0,001
35+	11.8%	10,1%	12,4%	13,4%	15,7%	+24.8%	< 0,001
Média (desvio padrão)	25,6 (6,6)	25,9 (6,4)	26,3 (6,5)	26,5 (6,6)	27,1 (6,6)	+5.9%	-
Mediana	25	25	26	26	26	+4,0%	-
Cor da pele							
Branca	69,5%	69,4%	66,1%	67,0%	76,5%	+10,1%	< 0,001
Parda	18,3%	20,6%	22,3%	22,6%	15,0%	-18,0%	0,156
Preta	12,2%	9,9%	11,7%	10,3%	8,5%	-30,3%	< 0,001
Viviam com marido/companheiro	82,8%	83,2%	85,8%	83,6%	85,1%	+1.6%	0,034
Escolaridade da mãe (anos)							
0	0,9%	0,4%	0,0%	0,1%	0,1%	-88,9%	< 0,001
1 a 4	11,7%	7,6%	6,0%	3,3%	4,1%	-65,0%	< 0,001
5 a 8	36,1%	37,2%	33,6%	33,4%	27,3%	-24,4%	< 0,001
9 a 11	41,9%	44,5%	44,7%	39,8%	46,9%	+11,9%	0,139
12+	9,4%	10,3%	15,6%	23,5%	21,6%	+129,8%	< 0,001
Média (desvio padrão)	8,6 (3,5)	9,0 (3,2)	9,5 (3,3)	10,1 (3,6)	10,5 (3,9)	+22,1%	-
Mediana	9	10	10	11	11	+22,2%	-
Exerceram trabalho remunerado durante a gravidez (n = 12.136)	37,4%	42,8%	43,6%	45,9%	42,6%	+13,6%	< 0,001
Renda familiar mensal em salários mínimos (SM) (n = 12.184)							
< 1	12,4%	9,6%	3,4%	5,9%	9,9%	-20,2%	< 0,001
1,0 a 1,9	33,4%	37,1%	29,1%	31,4%	34,6%	+3,6%	0,295
2,0 a 3,9	34,7%	34,2%	40,2%	40,6%	40,1%	+15,6%	0,077
≥ 4,0	19,6%	19,1%	27,3%	22,1%	15,4%	-21,4%	< 0,001
Média (desvio padrão) em SM	2,9 (3,2)	3,2 (3,9)	3,4 (2,9)	3,2 (3,1)	2,6 (2,3)	-10,3%	-
Mediana em SM	2,1	2,2	2,6	2,3	2,0	-4,8%	-
Número de moradores no domicílio							
Média (desvio-padrão)	3,7 (1,8)	3,5 (1,8)	3,4 (1,7)	3,3 (1,6)	3,5 (1,5)	-5,4%	-
Mediana	3	3	3	3	3	0,0%	-
Pessoa de maior renda desempregada (n = 12.136)	15,2%	10,5%	7,3%	16,8%	15,1%	-0,7%	0,008
Mulher como pessoa de maior renda	8,8%	11,1%	9,9%	11,5%	9,0%	+2,3%	0,169

Total	n	2.557	2.395	2.687	2.694	2314	
	%	20,2	18,9	21,2	21,3	18,3	

A Tabela 2 mostra queda de aproximadamente 50% na prevalência de tabagismo durante a gravidez e nos seis meses precedentes entre 2007 e 2019.

**Tabela 2:** Características reprodutivas, hábitos de vida e morbidade no período gestacional entre parturientes incluídas nos estudos perinatais de Rio Grande (RS), 2007–2019.

Característica		2007	2010	2013	2016	2019	Variação 2007–2019	p-valor tendência
Primíparas		39,5%	43,4%	47,3%	43,1%	38,1%	-3,5%	0,457
Paridade								
Média (desvio padrão)		2,1 (1,4)	2,0 (1,3)	1,8 (1,0)	2,2 (1,3)	2,1 (1,3)	0,0%	-
Mediana		2	2	2	2	2	0,0%	-
Se já teve algum filho nascido morto		3,5%	3,5%	1,7%	2,8%	2,2%	-37,1%	0,003
Referiram ocorrência de aborto (espontâneo e/ou induzido) no passado		19,2%	13,8%	15,3%	15,3%	15,2%	-20,8%	0,018
Fumaram pelo menos um cigarro por dia nos seis meses anteriores à gravidez		27,8%	26,3%	22,5%	15,1%	14,1%	-49,3%	< 0,001
Fumaram pelo menos um cigarro por dia durante pelo menos um dos trimestres da gestação		23,1%	20,8%	18,7%	12,7%	12,5%	-45,9%	< 0,001
Morbidade referida no período gestacional								
Hipertensão arterial sistêmica		18,0%	19,6%	19,6%	18,2%	12,5%	-30,6%	< 0,001
Diabete mellitus		2,9%	3,1%	5,1%	4,5%	9,3%	+220,7%	< 0,001
Anemia		51,7%	41,5%	36,6%	31,2%	37,8%	-26,9%	< 0,001
Depressão		16,9%	10,2%	9,9%	3,6%	2,2%	-87,0%	< 0,001
Corrimento vaginal patológico		52,0%	42,9%	43,4%	32,5%	21,7%	-37,5%	< 0,001
Hospitalizações maternas		13,4%	10,7%	8,9%	8,9%	5,3	-60,4%	< 0,001

Total	n	2.557	2.395	2.687	2.694	2314		
	%	20,2	18,9	21,2	21,3	18,3		

Na Tabela 3 , verifica-se que a assistência pré-natal melhorou substancialmente no período. Mais mulheres iniciaram o pré-natal ainda no primeiro trimestre da gestação, e o número de consultas também aumentou. A grande maioria das mães completou pelo menos seis consultas e realizou dois testes sorológicos para HIV, sífilis e urina. No entanto, ainda é baixo o índice de realização de exame citopatológico de colo uterino e clínico das mamas. Em 2019, o parto vaginal voltou a ser o mais comum. A ocorrência de episiotomia caiu de 71% para 19%, e o atendimento ao parto por médico foi praticamente universalizado. Por fim, 58% das parturientes passaram por todas as consultas de pré-natal, e 76% realizaram o parto no Sistema Único de Saúde (SUS).

**Tabela 3:** Assistência pré-natal e ao parto entre puérperas incluídas nos estudos perinatais de Rio Grande (RS), 2007–2019.

Característica		2007	2010	2013	2016	2019	Variação 2007–2019	p-valor tendência
Planejaram a gravidez		37,0%	36,0%	37,5%	39,4%	32.8%	-11.4%	< 0,001
Realizaram pré-natal		95,8%	95,5%	97,4%	98,5%	96,7%	+3.4%	< 0,001
Completaram seis ou mais consultas		72,5%	76,7%	83,5%	84,3%	85,7%	+18.2%	< 0,001
Número de consultas de pré-natal realizadas								
Média (desvio padrão)		7,4 (3,7)	7,7 (3,6)	8,3 (3,3)	8,2 (3,1)	8,7 (3,4)	10.8%	
Mediana		7	8	8	8	9	14.3%	-
Iniciaram as consultas no 1º trimestre		73,6%	78,3%	78,6%	79,4%	81,5%	+10,7%	< 0,001
Local de realização do pré-natal								
UBS		41,0%	33,5%	29,7%	36,4%	49,2%	+20,0%	< 0,001
Ambulatório		20,0%	24,3%	22,0%	20,7%	14,6%	-27,0%	< 0,001
Particular/Convênio		39,0%	42,1%	48,3%	42,9%	36,2%	-7,2%	0,225
Realizaram durante as consultas de pré-natal:								
2+ testes sorológicos para HIV		67,7%	61,4%	75,8%	84,4%	93,6%	+38,3%	< 0,001
2+ testes sorológicos para sífilis		27,0%	56,2%	73,5%	78,4%	87,4%	+223,7%	< 0,001
2+ exames qualitativos) de urina		70,8%	72,1%	76,8%	75,9%	85,0%	+117,9%	< 0,001
1+ ultrassonografia abdominal		92,3%	92,8%	94,9%	98,4%	93,3%	+1,1%	< 0,001
Realizaram exame citológico de colo uterino		37,5%	37,8%	45,7%	30,9%	57,6%	+53,6%	< 0,001
Tiveram o seio examinado		47,1%	56,2%	49,2%	39,9%	65,3%	+38,6%	< 0,001
Foram imunizadas contra tétano		78,5%	79,0%	82,5%	78,1%	85,4%	+8.8%	< 0,001
Receberam sulfato ferroso		59,0%	75,6%	78,8%	80,1%	81,1%	+37,5%	< 0,001
Iniciaram as consultas de pré-natal no primeiro trimestre, realizaram seis ou mais consultas e pelo menos dois testes de HIV, sífilis e qualitativos de urina (n = 8.828)		18,1%	40,2%	51,6%	37.4%	64,0%	+253,6%	< 0,001
Tipo de parto								
Vaginal		48,4%	43,4%	38,5%	45,8%	50,5%	+4,5%	0,069
Cesariana		51,6%	56,6%	61,5%	54,2%	49,5%	-4,1%	
Foi necessário uso de fórcipe		7,3%	9,2%	7,7%	4,7%	2,0%	-72,6%	< 0,001
Se foi submetida a episiotomia (n = 5.715)		70,9%	68,0%	60,2%	40,1%	19,4%	-70,9%	< 0,001
Tiveram o parto atendido por médico		85,3%	92,4%	95,3%	97,1%	98,8%	+15,8%	< 0,001
Fizeram parto no SUS		79,1%	75,9%	65,5%	76,2%	83,9%	+6,1%	0,001

Total	n	2.557	2.395	2.687	2.694	2314		
	%	20,2	18,9	21,2	21,3	18,3		

A Tabela 4 mostra que a grande maioria das crianças nasce com peso adequado (91%) e a termo (83%). Poucos recém-nascidos necessitam de hospitalização (6%), e menos de um quarto apresenta fator de risco importante (22%). Baixo peso ao nascer (< 2.500 g) e prematuridade (< 37 semanas de gestação) acometeram 9% e 17% dos recém-nascidos, respectivamente. Pouco mais da metade (56%) das mães deu entrada no hospital portando a Carteira da Gestante.

**Tabela 4:** Principais características dos recém-nascidos pertencentes aos estudos perinatais de Rio Grande (RS), 2007–2019.

Característica		2007	2010	2013	2016	2019	Variação 2007–2019	p-valor tendência
Sexo								
Masculino		51,2%	51,0%	52,4%	51,0%	51,0%	+0,4%	0,907
Feminino		48,8%	49,9%	47,6%	49,0%	49,9%		
Recém-nascidos de parto único		98,7%	98,3%	97,5%	98,3%	98,1%	-0,6%	0,166
Nasceram vivos		98,5%	99,1%	98,8%	99,1%	99,4%	+0,9%	0,005
Peso ao nascer (em gramas)								
≤ 2499		9,4%	8,9%	9,7%	8,8%	10,1%	+7,4%	0,533
2500–2999		22,8%	24,3%	23,0%	21,6%	20,8%	-8,8%	0,015
3000–3499		40,6%	39,2%	40,0%	40,7%	40,1%	-1,2%	0,866
3500–4999		21,5%	21,8%	21,9%	22,5%	23,1%	+7,4%	0,146
≥ 5000		5,7%	5,8%	5,4%	6,4%	5,8%	+1,8%	0,510
Média (desvio padrão) em gramas		3.172 (594)	3.184 (577)	3.171 (588)	3.203 (569)	3.190 (590)	+5,7%	-
Mediana		3.205	3.220	3.205	3.275	3.230	+7,8%	-
Idade gestacional (semanas) (n=10825)								
20–33,9		6,0%	5,2%	7,2%	5,4%	7,1%	+18,3%	0,210
34–36,9		12,2%	10,5%	11,4%	10,1%	10,0%	-18,0%	0,023
37–39,9		48,0%	49,9%	50,1%	48,3%	48,8%	+1,7%	0,917
40+		33,8%	34,3%	31,2%	36,1%	34,1%	+0,9%	0,474
Média (desvio padrão) em gramas		38,7 (3.1)	38.8 (3,0)	38,5 (3,1)	38,9 (3,0)	38,6 (3,2)	+0,1%	-
Mediana em gramas		39,1	39,1	40,3	39,3	39,3	-0,2%	-
Precisou ficar hospitalizada		6,4%	5,5%	7,0%	6,1%	6,1%	-4,7%	0,792
Nasceram com baixo peso, prematuros e/ou tiveram de ser hospitalizados (n = 10.825)		23,6%	21,4%	24,7%	20,6%	21,5%	-8,9%	0,075
Mães que portavam Carteira da Gestante quando foram hospitalizadas para o parto		54,7%	51,3%	59,1%	53,5%	60,8%	+11,2%	< 0,001

Total	n	2.557	2.395	2.687	2.694	2314		
	%	20,2	18,9	21,2	21,3	18,3		

## DISCUSSÃO

Os dados mostram melhora na condição socioeconômica das famílias e, principalmente, na assistência à gestação e ao parto no municipio de Rio Grande entre 2007 e 2019. Nesse período, as mães ganharam dois anos de escolaridade, aumentaram e mantiveram participação no mercado de trabalho, postergaram a idade da gravidez e abandonaram de forma expressiva o tabagismo. As crianças nasceram com boas condições de saúde, mas seguem convivendo com elevados índices de baixo peso ao nascer e, sobretudo, prematuridade. Apesar de, em 2016, alguns indicadores terem deixado de melhorar ou até mesmo terem piorado, a assistência recebida durante a gestação e o parto em 2019 é muito superior àquela observada em 2007.

Os partos na adolescência em Rio Grande caíram 35% no período, enquanto aumentaram 25% entre mulheres com 35 anos ou mais. No Brasil, o índice de parto na adolescência caiu de 36% em 2000 para 24% em 2017^15^ . Em Ribeirão Preto (SP), de 29,3% em 1997/1998 para 18,5% em 2010^7^ , e em Pelotas de 15,4% em 1982 para 14,6% em 2015^6^ . Em relação à proporção de partos entre mulheres com 35 anos ou mais, o aumento observado em Rio Grande é semelhante ao de Pelotas, passando de 9,9% em 1982 para 14,8% em 2015^6^ . No país como um todo, esse dado pouco se modificou. Em 1996, 9,9% das parturientes tinham 35 anos ou mais^16^ , número que chegou a 10,5% em 2011/2012^5^ e 11,8% em 2012/2013^17^ .

A diminuição na ocorrência de gravidez entre adolescentes, assim como o aumento entre mulheres de maior idade, pode ser atribuída principalmente ao maior acesso a serviços de saúde^17^ , ao aumento do nível de escolaridade^18^ , à queda na taxa de fecundidade^3^ e à maior inserção das mulheres no mercado de trabalho, sobretudo em Rio Grande, em decorrência da indústria naval^19^ .

Diversos estudos apontam melhora substancial na assistência à gestação e ao parto no Brasil nas últimas três ou quatro décadas^3 , 6 , 7 , 9^ . O divisor de águas para essa mudança foi o estabelecimento do SUS em 1989^20^ . Desde então, diversos programas foram implementados, com destaque para a atual Estratégia Saúde da Família (ESF)^21^ .

Em Rio Grande, o acesso ao pré-natal pouco se modificou no período, ficando em 97%, mas a realização de pré-natal considerado minimamente adequado aumentou de 18,1% em 2007 para 63,2% em 2019. Em Pelotas, este indicador passou de 41% em 1982 para 63% em 2015^6^ , e em São Luís, de 47,3% em 1997/1998 para 58,2% em 2010^8^ . No Brasil como um todo, o salto foi de 15%^17^ para 71,4%^5^ . No entanto, cabe observar que a comparação com outros estudos, no que se refere a esse indicador específico, fica prejudicada em virtude da utilização de critérios diferentes.

A melhoria observada em Rio Grande decorre da expansão da cobertura da atenção básica, que passou de 55,3% em 2007 para 87,5% em 2019. Esse crescimento se deveu ao aumento no número de equipes da ESF e maior oferta de serviços na periferia e área rural da cidade. No entanto, aumentar a proporção de mães com pré-natal adequado ainda é um grande desafio no município. Persiste também a necessidade de elevar a oferta de exame clínico das mamas, exame citopatológico de colo uterino, imunização contra tétano neonatal e suplementação com sulfato ferroso. Apesar de 80% de todas as gestantes iniciarem o pré-natal no primeiro trimestre e realizarem seis ou mais consultas, a cobertura para os procedimentos citados ficou ao redor de 60%, o que denota perda de oportunidade de intervenção.

A ocorrência de cesarianas aumentou de 51,6% em 2007 para 61,5% em 2013, mas depois caiu para 49,5% em 2019. Apesar de a queda em todo o período ser de apenas 4%, entre 2016 e 2019 essa redução foi de 20%, o que mostra clara tendência de redução do procedimento no município. No Brasil, tal ocorrência passou de 40,2% em 1995 para 55,5% em 2015^3^ . Em Pelotas, aumentou de 27,6% em 1982 para 64,9% em 2015^6^ , enquanto em São Luís foi de 34,1% em 1997/1998 para 47,5% em 2010^7^ .

A redução das cesarianas em Rio Grande, apesar da ausência de intervenções desencorajando o procedimento, decorre do fechamento parcial e temporário de um dos hospitais locais, que atendia toda a demanda do setor privado. Nele, a frequência de cesarianas era 50% maior em comparação com o hospital voltado exclusivamente aos pacientes do SUS. Vale destacar, porém, que a proporção de cesarianas no município é ainda três vezes superior à preconizada como razoável pela Organização Mundial da Saúde (OMS), incluindo aqui gravidezes de risco^22^ .

A ocorrência de episiotomia em Rio Grande caiu de 70,9% em 2007 para 19,4% em 2019. Não se encontrou um único estudo de base populacional avaliando tendência para episiotomia no Brasil, o que dificulta a comparação. O estudo Nascer no Brasil encontrou índice de 56,1% para o país como um todo, variando de 48,6% na região Norte a 69,2% no Centro-Oeste, e de 55,5% no setor público a 67,1% no setor privado^23^ .

Além de não haver evidencia clínica da necessidade de episiotomia, sua ocorrência leva a sangramentos, lesões na região perineal, trauma esfincteriano, incontinência fecal e prolongamento da dor no pós-parto, entre outras complicações^24^ . Apesar disso, a OMS sugere como aceitável uma ocorrência de até 10%^22^ . Os elevados índices observados no Brasil decorrem do fato de que os partos são realizados principalmente por médicos, cuja prática se caracteriza pelo excesso de intervenções obstétricas^25 , 26^ .

Assim como para cesarianas, não houve intervenção específica em Rio Grande voltada à redução das episiotomias. As mudanças ocorridas no período podem estar relacionadas à obrigatoriedade, estabelecida pelo Ministério da Saúde, de autorização da mulher para que esse procedimento seja realizado, à presença do acompanhante no pré-parto e à maior participação da área de enfermagem. O fechamento parcial do outro hospital, onde a frequência de episiotomia era maior, também deve ter contribuído para essa queda bastante acentuada.

As proporções de baixo peso ao nascer e de prematuridade em Rio Grande passaram de 9% e 18% em 2007 para 10% e 17% em 2019. Em Pelotas, a prevalência de baixo peso aumentou de 9% em 1982 para 10% em 2015, enquanto a de prematuridade saltou de 6% para 14%^26^ . Em São Luís, as prevalências de baixo peso ao nascer e prematuridade mantiveram-se praticamente estáveis entre 1997/1998 e 2010, em cerca de 8% e 13%, respectivamente^7^ . No Brasil como um todo, a ocorrência de baixo peso ao nascer e prematuridade manteve-se entre 1995 e 2015 em 8% e 11%, respectivamente^3^ .

Considerando as melhorias ocorridas desde a implementação do SUS em 1989 – expansão da rede básica de saúde, Estratégia Saúde da Família, Rede Cegonha e Programa Mais Médicos, entre outras iniciativas –, a única explicação possível para a ausência de queda (ou até mesmo elevação em algumas localidades) é o excesso de cesarianas^26^ . Em Rio Grande, a proporção de cesarianas entre mães do maior quintil de renda é de 74,4% contra 41,1% do pior quartil.

A prevalência de tabagismo antes e durante a gestação caiu cerca de 50% no período. Tendência semelhante foi observada em Pelotas (de 35,7% em 1982 para 16,5% em 2015) e em Ribeirão Preto (de 28,8% para 11,8%)^27^ . Em Pelotas, essa queda se deu principalmente entre mulheres brancas e de alta renda^28^ . No país como um todo, a queda foi de 15,6% em 2006 para 10,8% em 2014^29^ .

O tabagismo é prejudicial à saúde do feto porque restringe o crescimento intrauterino e aumenta as chances de prematuridade e baixo peso ao nascer^30^ . As políticas públicas antitabagismo devem priorizar mães de maior vulnerabilidade social. Caso medidas não sejam tomadas, o tabagismo continuará causando danos à saúde materno-infantil, com sequelas na vida adulta^28^ .

A portabilidade da Carteira da Gestante no momento do parto aumentou de 54,7% para 60,8% em Rio Grande. Nenhum dos estudos já citados publicou dados sobre esse indicador. O estudo Nascer no Brasil encontrou prevalência de posse quando da admissão hospitalar de 74,6% para o Brasil como um todo, variando de 46% na região Centro-Oeste a 83,6% na região Sul^4^ .

A posse da Carteira da Gestante costuma ser baixa porque se trata de documento pouco valorizado. Esforços precisam ser feitos para conscientizar as mães sobre a necessidade de levar essa carteira a todas as consultas, assim como os profissionais devem preencher o documento de forma adequada e completa, o que não vem ocorrendo^31^ . A utilização correta da Carteira da Gestante otimizaria a oferta de cuidados à gestante e ao recém-nascido.

A piora da renda familiar observada a partir de 2016 se deve basicamente à redução da oferta de empregos no setor naval de Rio Grande. A montagem de plataformas de petróleo gerou, entre 2007 e 2008 e 2015 e 2016, cerca de 30 mil empregos diretos e indiretos na região^19^ , ocupando toda a mão de obra excedente em Rio Grande e municípios vizinhos. Em 2019, no entanto, o mesmo setor gerava pouco mais de uma dezena de empregos.

Os estudos perinatais de Rio Grande talvez sejam a única fonte de dados primários coletados com periodicidade regular, em intervalos curtos e por período relativamente longo, em um município brasileiro de porte médio. Utilizou-se a mesma metodologia nos cinco anos de investigação, e a taxa de respondentes foi bastante alta, incluindo quase a totalidade de nascimentos tanto da área urbana como rural do município. A única limitação do estudo é o fato de se basear quase que exclusivamente em informações fornecidas pela mãe, havendo, portanto, possibilidade de viés de memória. Isso, no entanto, não inviabiliza os resultados, visto que os estudos aqui utilizados para comparação apresentam esse mesmo problema.

A série de inquéritos mostrou que praticamente todos os indicadores de assistência à gestação e ao parto vêm melhorando no município de Rio Grande. Não há dúvida, pelo menos em termos quantitativos, que os cuidados recebidos em 2019 são superiores aos de 2007. No entanto, há de se ressaltar que a universalização da oferta de cuidados adequados, tanto durante o pré-natal quanto no momento do parto, ainda estão muito aquém do desejado. Evidências ainda apontam intervenções desnecessárias e oportunidades perdidas. A equipe local de saúde precisa envidar esforços no sentido de assegurar à gestante todos os cuidados necessários. Otimizar a oferta destes cuidados é essencial para reduzir a morbimortalidade materno-infantil no município. Por fim, destaca-se a importância do SUS, local de realização da maioria das consultas de pré-natal e partos. Fortalecê-lo é uma questão de justiça social em tempos tão obscuros e incertos como os que o Brasil vem atravessando.
